# Editorial: Systems biology, women in science 2021/22: Data and model integration

**DOI:** 10.3389/fsysb.2023.1134055

**Published:** 2023-01-31

**Authors:** María Rodríguez Martínez, Angelyn Lao, Leda Torres

**Affiliations:** ^1^ IBM Research—Zurich, Rüschlikon, Switzerland; ^2^ Department of Mathematics and Statistics De La Salle University, Manila, Philippines; ^3^ Laboratorio de Citogenética, Instituto Nacional de Pediatría (México), Mexico City, Mexico

**Keywords:** women in science, STEM—Science Technology Engineering Mathematics, model integration, data, systems biology

Despite recent progress in encouraging and retaining talented women in science, technology, engineering, and mathematics (STEM) careers, women still face stiff penalties in the academic world. Research shows that women receive less funding, awards, teaching scores, invitations to speak at conferences, and citations than male colleagues (Berggren et al., 2022; Ainslie, 2022). To facilitate the success of our female colleagues and trainees in academia, this Research Topic aimed to highlight the work of women in Systems Biology, with a special focus on showcasing research on Data and Model Integration. It spans advances in theory, methodology, and experimental work with applications to biologically compelling problems.

This Research Topic includes six original research articles, one perspective article and one technology and code article, with the participation of 41 authors from 10 countries: Colombia, France, Germany, Greece, Ireland, Mexico, Netherlands, Philippines, Switzerland, and the United Kingdom. We have a total of 7,493 views as of 9 January 2023. Overall, we were very pleased by the quality of the submissions we received in response to the call.

In the Model Integration area, Connolly and colleagues presented a methodology for pandemic modelling motivated by the current COVID-19 outbreak with the title “From Epidemic to Pandemic Modelling” (Connolly et al.) Pandemic models are important to design effective control measures, such as travel or quarantine restrictions. Here, the authors proposed a methodology for systematically extending epidemic models to multilevel and multiscale spatiotemporal pandemic models that integrate information about geography and travel connections. PetriNuts, a publicly available web-based platform, supports model construction, simulation, and output visualization. It also enables deterministic, stochastic and hybrid simulation, as well as structural and behavioural analysis.

Flores-Garza and co-authors published “Mathematical Model of the Immunopathological Progression of Tuberculosis,” an elegant model to understand tuberculosis, a worldwide persistent infectious disease caused by the bacteria *Mycobacterium tuberculosis* (Flores-Garza et al.). A mechanistic mathematical model integrates multiple *in vivo* and *in vitro* data from immunohistochemical, serological, molecular biology, and cell count assays. Ordinary differential equations (ODEs) were used to describe the regulatory interplay between the cell phenotypic variation and the inflammatory microenvironment. The model can predict disease outcomes for different mouse genotypes and simulate the interaction between host and pathogen genotypes. In doing so, it provides a powerful tool to test the effect of host-pathogen interaction alterations on infection outcomes. These *in silico* experiments can lead to future experimentation and help reduce the number of *in vivo* experiments.

Freiesleben and co-authors focused on unravelling the molecular players involved in modulating DNA-damage response (DDR) after exposure to ionizing radiation (IR). Their paper “A workflow for the creation of regulatory networks integrating miRNAs and lncRNAs associated with exposure to ionizing radiation using open-source data and tools” (Freiesleben et al.) focused specifically on the role of non-coding RNAs in DDR. The authors integrated transcriptomics data and regulatory information to create networks that capture the interplay between genes, transcription factors, microRNAs, and long non-coding RNAs following IR. Armed with this workflow, they identified differentially expressed genes and constructed regulatory networks that enabled them to investigate the regulatory mechanisms involving microRNAs and long non-coding RNAs in the molecular response to IR.

Another contribution to this Research Topic was the original research from Tejero and collaborators, “Multi-Scale Modeling Recapitulates the Effect of Genetic Alterations Associated with Diffuse Large B-Cell Lymphoma in the Germinal Center Dynamics” (Merino Tejero et al.). Focusing on diffuse large B-cell lymphoma, an aggressive lymphoma that arises in the germinal centres (GCs), the follicles where B cells replicate and diversify their immunoglobulin genes, the authors presented a multiscale model that combined ODEs to capture gene regulatory dynamics and agent-based models to account for cellular interactions. Using this model, the authors investigated the effect of genetic alterations on 3 transcription factors that are essential to regulate the GC reaction and B-cell differentiation. The multiscale model turns out to be a very valuable tool to investigate the onset of diffuse large B-cell lymphoma and investigate new therapeutic strategies.

Espina and co-authors focused on investigating the correlations between cerebral glucose metabolism and Alzheimer’s disease (AD), a neurodegenerative disorder that causes drastic brain structural and cognitive atrophy. As several studies have proposed the use of ketogenic interventions to improve cognitive function in AD patients, the authors developed a mathematical model of cerebral glucose and ketone bodies metabolism to investigate the effect of glucose hypometabolism on both healthy ageing and AD patients. In their article “Modelling the Effects of Medium-Chain Triglycerides on Cerebral Ketone Body Metabolism” (Espina et al.), they suggested that common ketogenic interventions can boost the brain’s energy source availability and improve the cognitive performance of both AD and mild cognitive impaired patients.

In the Data analysis area, Angarita-Rodríguez and colleagues integrated transcriptomic and proteomic data into a human astrocyte genomic scale metabolic model. Their article is entitled “Multi-Omics Integrative Analysis Coupled to Control Theory and Computational Simulation of a Genome-Scale metabolic Model Reveal Controlling Biological Switches in Human Astrocytes Under Palmitic Acid-Induced Lipotoxicity” (Angarita-Rodríguez et al.), and described a combination of metabolic flux analysis and control theory to identify the reactions that govern the astrocytic system. The model not only enables the investigation of the mechanisms underpinning metabolic regulation but can also direct future experimental work in neurodegenerative diseases.

Zerrouk and colleagues presented “A Mechanistic Cellular Atlas of Rheumatic Join” (Zerrouk et al.), where they developed a mechanistic mapping of the molecular pathways and cellular crosstalks underpinning Rheumatoid Arthritis (RA), an autoimmune disease associated with synovial inflammation, bone erosion and cartilage destruction in the patients’ joints. Using a combination of prior knowledge extracted from the literature and pathway databases, and mining of omics data, the authors developed a RA multicellular atlas of the rheumatic joint that recapitulates existing knowledge related to the disease’s onset, progression, and pathogenesis. The atlas is freely accessible and compatible with the SBGN standards, one of the most common systems biology formats. Furthermore, it enables user-directed navigation, data overlay, as well as gene set enrichment analysis, pathway export, and drug query.

Finally, Pantziri and Klapa published a perspective article, “Standardization of Human Metabolic Stoichiometric Models: Challenges and Directions” (Pantziri et al.), where they presented an overview of existing human metabolic models. The authors described current human metabolic stoichiometric models, identified the standardization of the reconstruction methods, representation formats and model repositories as a major challenge that currently limits model selection and comparison, and proposed potential solutions. They also highlighted that standardization is essential for the successful integration of metabolomic and metabolic flux data with other omics and biological data.

On balance, we were very pleased with the depth, breadth, and diversity of the published papers. Most of the first authors are doctoral students who will undoubtedly be brilliant young researchers in the near future. The corresponding authors are mostly female researchers in the process of consolidation and we are pleased that they chose to present their work in this research topic. We hope that this initiative and collection helps highlight the outstanding work our female colleagues are producing in systems biology, and we look forward to continuing to encourage the development of female talent in STEM careers.
